# Shared and unique neural circuitry underlying temporally unpredictable threat and reward processing

**DOI:** 10.1093/scan/nsab006

**Published:** 2021-01-15

**Authors:** Milena Radoman, Lynne Lieberman, Jagan Jimmy, Stephanie M Gorka

**Affiliations:** Department of Psychiatry, University of Illinois-Chicago, Chicago, IL 60612, USA; Graduate Program in Neuroscience, University of Illinois-Chicago, Chicago, IL 60612, USA; Road Home Program, Rush University Medical Center, Chicago, IL 60612, USA; Department of Psychiatry, University of Illinois-Chicago, Chicago, IL 60612, USA; Department of Psychiatry and Behavioral Health, Ohio State University, Columbus, OH 43205, USA

**Keywords:** unpredictable threat, unpredictable reward, NPU, fMRI

## Abstract

Temporally unpredictable stimuli influence behavior across species, as previously demonstrated for sequences of simple threats and rewards with fixed or variable onset. Neuroimaging studies have identified a specific frontolimbic circuit that may become engaged during the anticipation of temporally unpredictable threat (U-threat). However, the neural mechanisms underlying processing of temporally unpredictable reward (U-reward) are incompletely understood. It is also unclear whether these processes are mediated by overlapping or distinct neural systems. These knowledge gaps are noteworthy given that disruptions within these neural systems may lead to maladaptive response to uncertainty. Here, using functional magnetic resonance imaging data from a sample of 159 young adults, we showed that anticipation of both U-threat and U-reward elicited activation in the right anterior insula, right ventral anterior nucleus of the thalamus and right inferior frontal gyrus. U-threat also activated the right posterior insula and dorsal anterior cingulate cortex, relative to U-reward. In contrast, U-reward elicited activation in the right fusiform and left middle occipital gyrus, relative to U-threat. Although there is some overlap in the neural circuitry underlying anticipation of U-threat and U-reward, these processes appear to be largely mediated by distinct circuits. Future studies are needed to corroborate and extend these preliminary findings.

## Introduction

The adaptive ability to predict and effectively prepare for the possible (that is uncertain) future outcomes, both positive and negative in valence, is essential for the well-being and self-preservation of organisms. Empirical research to date has shown that under uncertain conditions, both animals and humans exhibit sustained vigilance and apprehensive responding, particularly when there is a potential for an aversive outcome (Prokasy, 1956; Cantor and LoLordo, 1970; Seligman and Meyer, 1970; Imada and Nageishi, 1982; Herry *et al.*, 2007; Bar-Anan *et al.*, 2009; Grupe and Nitschke, 2011; Davies and Craske, 2015; Dieterich *et al.*, 2016; Ran *et al.*, 2016; Anselme and Güntürkün, 2018). These anticipatory responses to uncertain events and situations with valenced outcomes (threatening or rewarding) are notably aberrant in clinical populations with anxiety, depressive, obsessive-compulsive and other disorders (Greco and Roger, 2003; Dugas *et al.*, 2004; Gentes and Ruscio, 2011; Olino *et al.*, 2011; Carleton *et al.*, 2012; Baskin-Sommers and Foti, 2015; Carleton, 2016; Nelson *et al.*, 2016; Shihata *et al.*, 2016). Increasing our understanding of the neural mechanisms underlying response to uncertainty may therefore shed light on pathophysiology of multiple psychopathologies.

Most of the research regarding uncertainty has been focused on examining reactivity to unpredictable threats, which come in many forms including uncertain timing, intensity, frequency and/or duration (Shankman *et al.*, 2011; Schmitz and Grillon, 2012; Bradford *et al.*, 2013; Daldrup *et al.*, 2015; Davies and Craske, 2015; Quelhas Martins *et al.*, 2015; Bennett *et al.*, 2018). In particular, uncertainty associated with not knowing when an aversive event may occur (temporal unpredictability) is a potent elicitor of sustained anxiety and hypervigilance across species (Sudha and Pradhan, 1993; Grillon *et al.*, 2004, 2006; Herry *et al.*, 2007; Seidenbecher *et al.*, 2016).

One of the most common paradigms used to measure response to temporally unpredictable threat in humans is the NPU task, which compares the anticipatory responses to three within-subject conditions—no threat (N; participants are safe from threat), predictable threat (P; threat is signaled by a predictable warning cue) and unpredictable threat (U; threat is unsignaled; Schmitz and Grillon, 2012; Grupe and Nitschke, 2013; Ferry and Nelson, 2020). NPU studies of healthy individuals have found that unpredictable negative events (e.g. shocks, aversive tones or images) elicit stronger psychophysiological responses than predictable ones, as evidenced by an increased startle eyeblink potentiation, a somatic marker of aversive responding (Grillon *et al.*, 2004, 2006; Schmitz *et al.*, 2011; Bach *et al.*, 2015; Nelson *et al.*, 2015; Schroijen *et al.*, 2016).

In order to elucidate the neural circuitry underlying heightened reactivity to unpredictable threat, studies have begun to employ variants of the NPU task during functional magnetic resonance imaging (fMRI). To date, studies have identified a specific frontolimbic circuit that may become engaged during the processing of aversive stimuli (Bach and Dolan, 2012; Grupe and Nitschke, 2013; Fox and Shackman, 2019; Hur *et al.*, 2020). This circuit is comprised of regions such as the amygdala, insula, bed nucleus of the stria terminalis, orbitofrontal cortex (OFC), anterior cingulate cortex (ACC) and the dorsolateral, ventrolateral and ventromedial prefrontal cortices (Herry *et al.*, 2007; Grupe and Nitschke, 2013; Shankman *et al.*, 2014; Tovote *et al.*, 2015; Goode *et al.*, 2019). Of these regions, insula appears to be particularly involved in responding to uncertainty, with the anterior agranular region of the insular cortex (anterior insula, AIC) playing a critical role in the anticipation of unpredictable aversiveness (Clark *et al.*, 2008; Craig, 2009; Khalsa *et al.*, 2009; Sarinopoulos *et al.*, 2010; Gu *et al.*, 2013; Shankman *et al.*, 2014). Evidence indicates that the AIC integrates information about the internal and external states to produce interoceptive awareness and generate anticipatory emotional responses to future events (Craig, 2011).

Interestingly, the AIC has recently also been implicated in processing of uncertain or unpredictable rewards. For example, using single-unit recordings, Mizuhiki *et al.* (2012) examined neural activity during stochastic reward delivery and found that dynamics of neuronal population activity in the AIC was modulated as a function of reward outcome uncertainty (i.e. whether a trial was rewarded or not). A separate study done in rodents found that magnitude and temporal dynamics of neuronal activity in the AIC encoded reward probability (i.e. the likelihood that a trial would be rewarded; Jo and Jung, 2016). In addition, a recent human fMRI study from our lab found increased activation in the bilateral AIC during the anticipation of unpredictable monetary rewards of varying magnitudes (Gorka *et al.*, 2016). Therefore, the activity in the AIC appears to be modulated by unpredictable rewards as well.

The AIC is also preferentially interconnected with the OFC, ACC and the ventral striatum (Mesulam and Mufson, 1982; Augustine, 1996; Chikama *et al.*, 1997), brain regions implicated in processing of information about uncertain outcomes (Critchley *et al.*, 2001; O’Doherty *et al.*, 2001; Dreher *et al.*, 2006; Yu *et al.*, 2011; Li *et al.*, 2016; Monosov, 2017; O’Neill and Schultz, 2018). Of these regions, the ventral striatum has emerged as a key node involved in reward anticipation (Berridge and Robinson, 1998, 2003; Diekhof *et al.*, 2012; Bartra *et al.*, 2013; Oldham *et al.*, 2018), particularly when the rewards occurred unexpectedly or were uncertain (Mirenowicz and Schultz, 1994; Apicella *et al.*, 1997; Schultz, 1998). Related to this, accumulating evidence suggests that activity of the striatal dopamine system reflects the anticipated reward magnitude, probability or delay within various behavioral contexts ranging from classic Pavlovian conditioning paradigms to widely used instrumental paradigms such as the monetary incentive delay (MID) task (Knutson *et al.*, 2000, 2001; Satoh *et al.*, 2003; Morris *et al.*, 2004; Tobler *et al.*, 2005; Dreher *et al.*, 2006; Preuschoff *et al.*, 2006; Kobayashi and Schultz, 2008; Hart *et al.*, 2015).

It is worth noting, however, that the experimental methodologies used to test neural response to reward uncertainty in humans, such as the MID task, often conflated explicit manipulations of multiple parameters of uncertain reward (e.g. probability and magnitude) with the implicit temporal unpredictability of the reward (i.e. when the reward may be delivered). Such approach made it difficult to examine the neural correlates of temporally unpredictable reward well separated from the coding of other reward parameters. This gap in the literature is noteworthy, given that temporal unpredictability influences behavior not only during the anticipation of aversive stimuli (as previously described), but also during the anticipation of appetitive stimuli (Mirenowicz and Schultz, 1994; Galtress *et al.*, 2012; Bermudez and Schultz, 2014). In this regard, it is critical to examine whether these two processes are mediated by overlapping or distinct neural systems, given that disruptions within these systems may lead to maladaptive anticipatory response to uncertainty (as a broadly defined construct), which is central to many clinical disorders.

To our knowledge, no study has run a direct, within-subject comparison of the neural circuitry underlying response to temporally unpredictable threat and reward. In addition, the paradigmatic differences between the tasks used to examine neural response to unpredictable threat (e.g. NPU) and reward (e.g. MID) represent a major confound when comparing the neural systems underlying these respective processes (e.g. blocked *vs* event-related design; passive *vs* button press). In order to fully assess the integrity of neural systems that signal temporally unpredictable threats and rewards, it is critical to employ experimental paradigms with little or no learning component, decision-making or active participation needed. The goal of the present study was to therefore examine the shared and unique neural correlates of temporally unpredictable threat and reward processing in a sample of young adults using fMRI variants of the well-validated NPU paradigm, in which temporal unpredictability of threat (i.e. mild electric shock) and reward (i.e. monetary incentive) was manipulated. The two tasks were specifically designed for the purposes of this study and were therefore analogous.

## Methods

### Participants

Participants (total *N* = 159) were taken from two samples recruited from the community as part of larger investigations on abnormal reactivity to uncertain stimuli (threat and reward) in relation to psychopathology. Participants were recruited via advertisements posted in the Chicago community, local psychiatric clinics and nearby college campuses. Demographic characteristics of the individual and pooled samples are listed in Table 1. General exclusion criteria included any major medical or neurological illness, psychosis, active suicidal ideation, deafness, traumatic brain injury, psychotropic medication use within the past four months, contraindications for fMRI, pregnancy, positive urine drug screen for illicit substances (including tetrahydrocannabinol, cocaine, amphetamine, morphine, phencyclidine, barbiturates, benzodiazepines, MDMA, oxycodone and buprenorphine) or breathalyzer test. Psychopathology was assessed via the Structured Clinical Interview for DSM-5 Disorders (First *et al.*, 2015), in person, by trained assessors and supervised by a clinical psychologist.

**Table 1.** Participant demographics and baseline clinical characteristics

	Sample 1 (*N* = 88)	Sample 2 (*N* = 71)	Pooled (*N* = 159)
Demographics			
Age (years)	18.5 (0.7)_a_	24.2 (2.9)_b_	21.0 (3.5)
Sex (% female)	67.0%_a_	49.3%_b_	59.1%
Ethnicity (% Hispanic)	35.2%_a_	31.0%_a_	33.3%
Education level (years)	12.9 (1.3)_a_	16.1 (1.9)_b_	14.3 (2.3)
Race			
White	60.2%_a_	57.7%_a_	59.1%
Black	8.0%_a_	4.2%_a_	6.3%
Asian	8.0%_a_	14.1%_a_	10.7%
American Indian or Alaskan Native	4.5%_a_	1.4%_a_	3.1%
Biracial, other or unknown	19.4%_a_	22.5%_a_	20.8%
SCID diagnoses			
Current major depressive disorder	5.7%_a_	6.9%_a_	6.3%
Current generalized anxiety disorder	5.7%_a_	9.7%_a_	7.5%
Current social anxiety disorder	12.6%_a_	1.4%_b_	7.5%
Current panic disorder	2.3%_a_	0.0%_b_	1.3%
Current specific phobia	1.1%_a_	1.4%_a_	1.3%
Current post-traumatic stress disorder	3.4%_a_	1.4%_a_	2.5%
Current eating disorder	0.0%_a_	2.6%_b_	1.3%
Current alcohol use disorder	0.0%_a_	39.1%_b_	19.5%
Lifetime major depressive disorder	34.5%_a_	26.4%_b_	30.8%
Lifetime generalized anxiety disorder	9.2%_a_	15.3%_a_	11.9%
Lifetime social anxiety disorder	16.1%_a_	2.8%_b_	10.1%
Lifetime panic disorder	5.7%_a_	6.9%_a_	6.3%
Lifetime-specific phobia	2.3%_a_	1.4%_a_	1.9%
Lifetime post-traumatic stress disorder	10.3%_a_	2.8%_b_	6.9%
Lifetime eating disorder	0.0%_a_	5.6%_b_	2.5%
Lifetime alcohol use disorder	0.0%_a_	56.6%_b_	28.3%
Substance use			
No. of drinks per week in the past month	0.7 (2.0)_a_	8.1 (6.7)_b_	4.1 (6.0)
No. of binges in the past month	0.1 (0.3)_a_	2.1 (2.7)_b_	1.0 (2.1)
Daily cigarette smoker (yes/no)	0.0%_a_	0.0%_a_	0.0%
No. of cigarettes smoked in the past month	1.3 (5.6)_a_	0.2 (1.7)_b_	0.8 (4.3)
Used cannabis in the past month (yes/no)	20.7%_a_	11.1%_b_	16.4%
No. of times used cannabis in the past month	0.7 (3.4)_a_	0.2 (0.6)_b_	0.5 (2.5)
Used other illicit drugs^a^ in the past month (yes/no)	2.7%_a_	4.6%_b_	3.8%
No. of times used other illicit drugs in the past month	0.07 (0.08)_a_	0.04 (0.27)_b_	0.03 (0.02)
Clinical variables			
AUDIT	3.0 (3.1)_a_	7.0 (4.7)_b_	4.8 (4.3)
IDAS-II General Depression	41.0 (11.9)_a_	35.0 (11.0)_b_	38.3 (11.8)
IDAS-II Anxiety	8.6 (2.6)_a_	6.9 (1.5)_b_	7.8 (2.3)

*Note*: Means (and s.d.) or percentages with different subscripts across rows were significantly different in pairwise comparisons (*P < *0.05, chi-square test for categorical variables and Tukey’s honestly significant test for continuous variables).

AUDIT, Alcohol Use Disorders Identification Test; IDAS-II, Inventory for Depression and Anxiety Symptoms-II; SCID, Structured Clinical Interview for DSM-5.

a Other illicit drugs refers to any illicit drug other than cannabis (e.g. cocaine, heroin and nonmedical prescription medications).

#### Sample 1.

Due to the aims of the larger study (not yet published), young adults were required to have had minimal alcohol exposure (i.e. self-reported consuming >1 but <100 standard alcoholic drinks in their lifetime), but be at risk for the onset of alcohol abuse by virtue of affiliating with risky peers and having access to alcohol. Participants were also required to be between the ages of 17 and 19. Study-specific exclusion criteria included lifetime history of alcohol or substance use disorder (SUD). A total of 109 individuals met inclusionary criteria; however, 18 were excluded due to missing/poor-quality fMRI data and 3 participants were excluded due to difficulty maintaining wakefulness, thus yielding a final sample of 88 individuals.

#### Sample 2.

As part of the aims of the larger study (Gorka *et al.*, 2019), participants were required to either have no personal or family history of alcohol use disorder (AUD) or meet the criteria for AUD within the past two years. Both groups were otherwise matched on the rates of other internalizing disorders. Participants were also required to be between 21 and 30 years old. Study-specific exclusion criteria included lifetime moderate or severe SUD (other than alcohol and nicotine). A total of 82 individuals met inclusionary criteria; however, 11 were excluded due to missing/poor quality fMRI data, thus yielding a final sample of 71 individuals.

Of note, poor fMRI data quality was defined in terms of excessive motion (i.e. >3 mm displacement in any direction) and/or presence of scanning artifacts.

Both studies took place at the University of Illinois at Chicago and were approved by the University Institutional Review Board. All participants provided written informed consent after review of the respective study protocols and were monetarily compensated for their time.

### Study procedure and fMRI tasks

Participants completed an initial screening and orientation visit during which they provided written informed consent and completed a clinical interview and battery of self-report questionnaires. During a separate fMRI visit, participants completed the following two complementary fMRI tasks designed to assess separate anticipatory processes (i.e. anticipating threat and reward). The tasks were presented in a counterbalanced order. For both studies, individuals were instructed to abstain from drugs and alcohol at least 24 h prior to the lab assessments, which was verified via breath alcohol and urine screens.

#### NPU threat task.

The fMRI threat task and laboratory procedures described here have been used previously by our group (Lieberman *et al.*, 2017; Gorka *et al.*, 2019). Briefly, each participant had two shock electrodes placed on their left foot in order to minimize movement and potential scan artifacts. Next, a shock work-up procedure was completed to determine the level of shock intensity that participants described as ‘highly annoying but not painful’ (between 1 and 5 mA). Ideographic shock levels were used to ensure equality in perceived shock aversiveness (Rollman and Harris, 1987). The shock stimuli lasted 400 ms and were delivered using a Biopac MP150 with an STM100C module (Biopac Systems, Inc., Goleta, CA) connected to a 200 V maximum stimulus isolation unit (STMISOC, Biopac System, Inc., Goleta, CA). Task stimuli were administered using Presentation software package (Neurobehavioral Systems, Inc., Albany, CA).

**Fig. 1.**
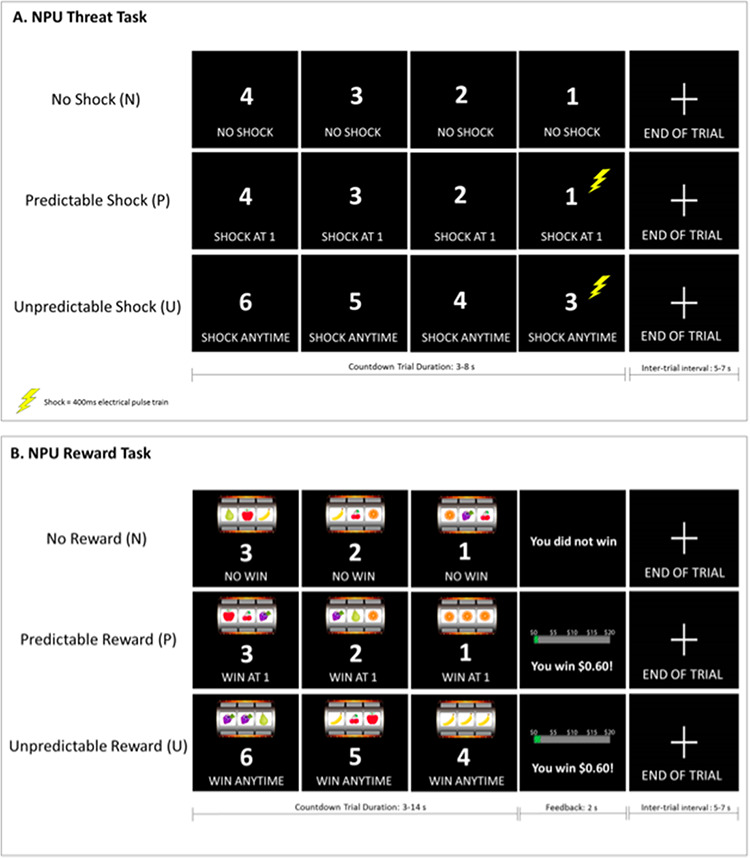
Illustrations of the NPU (A) threat and (B) reward tasks administered during scan.

To examine the neural correlates of temporally unpredictable threat, we used a modified version of the original NPU-threat task developed by Grillon and colleagues (Figure 1A; Schmitz and Grillon, 2012). The task included three, within-subject conditions: no shock (N), predictable shock (P) and unpredictable shock (U). During each condition, participants viewed a numeric countdown that ranged between 3 and 8 s, jittered (*M *= 5 s). Text at the bottom of the computer monitor informed participants of the current condition. During N trials, no shocks were delivered and the text read ‘No Shock’. During P trials, participants received a shock only when the countdown reached ‘1’ and the text read ‘Shock at 1’. During U trials, participants received a shock at random, regardless of the number on the screen and the text read ‘Shock at Anytime’. Following each countdown, individuals saw a fixation cross for 5–7 s, jittered (*M *= 6 s). N, P and U countdowns were presented in blocks of 6, and each condition/block was administered in a randomized order (counterbalanced) 6 times over the course of two runs. Participants received 10 electric shocks during P and 10 electric shocks during U, during each run. The rate of ‘Shock at 1’ during the P condition was 60%, consistent with the NPU version used by Grillon and colleagues (Schmitz and Grillon, 2012).

#### NPU reward task.

In order to examine the neural correlates of temporally unpredictable reward, our lab developed an analogous NPU reward task similar in its design and timing to the NPU threat task. Before the task, participants were told that they would be playing a slot machine game, similar to the one they would see at a casino, and that they had the chance to win up to $20 in cash. The task itself was a computerized, passive slot machine paradigm with three within-subject conditions: no reward (N), predictable reward (P) and unpredictable reward (U). The reward was a monetary prize of $0.60. During each condition, participants viewed a numeric countdown that ranged between 3 and 14 s, jittered (*M* = 8 s), and three reels of fruit, which ‘spun’ simultaneously for the duration of the countdown and then ‘landed’ on a result at the same time (Figure 1B). Text at the bottom of the computer monitor informed participants of the current condition. During N trials, no reward was delivered (i.e. the reels landed on three different fruits) and the text read ‘No Win’. During P trials, participants received a reward only when the countdown reached ‘1’ (i.e. the reels landed on three identical fruits) and the text read ‘Win at 1’. During U trials, participants received a reward at random (i.e. when the reels landed on three identical fruits), regardless of the number on the screen and the text read ‘Win at Anytime’. A feedback screen notified participants whether they won money or not during that trial and indicated their cumulative total winnings at that point. Following the feedback screen, individuals saw a fixation cross for 5–7 s, jittered (*M *= 6 s). N, P and U countdowns were presented in blocks of 4, and each condition/block was administered in a randomized order (counterbalanced) 6 times over the course of two runs. Participants were told before the game that the reward probabilities were random. Unbeknownst to the participants, the game was rigged to ensure that, consistent with the NPU threat task, 60% of the P and U trials resulted in a win, per each run.

### fMRI data collection and processing

fMRI was performed on a 3.0 Tesla GE MR 750 scanner (General Electric Healthcare; Waukesha, WI) using an 8-channel phased-array radio frequency head coil. A standard T2-sensitive gradient-echo echoplanar imaging sequence was used (2 s repetition time (TR); 22.2 ms echo time (TE); 90° flip; 64 × 64 matrix; 22 cm FOV; 44 axial slices; 3.44 × 3.44 × 3.0 mm voxels; 308 volumes per run). Structural scans were obtained with a 3D BRAVO pulse sequences with the following parameters: flip angle 13°, inversion time 450 ms, field of view 22 × 22 cm, matrix size 256 × 256, slice thickness 1 mm and 182 axial slices of the whole brain.

Statistical Parametric Mapping software (SPM12, Wellcome Department of Imaging Neuroscience, London, UK) was used to perform conventional preprocessing steps. Images were spatially realigned to correct for head motion, slice-time corrected (44 slices, TR = 2, TA = 2, slice order: ascending interleaved, reference slice 21), spatially normalized to Montreal Neurological Institute (MNI) space using the participants’ T1 structural image (default settings), resampled to 2 mm^3^ voxels and smoothed with an 8 mm^3^ kernel to minimize noise and residual differences in gyral anatomy. The general linear model was applied to the time series, convolved with the canonical hemodynamic response function and with a 128 s high-pass filter. Condition effects for U, P and N anticipation were separately estimated at each voxel for each subject. For each condition, *only* the countdowns prior to the shock (or reward), or prior to trial termination in instances where there was no shock (or no reward), were modeled. Importantly, number of data points (i.e. TRs/repetition times) was the same across the three conditions (N, P and U). Movement parameters obtained during realignment were included in the model as regressors-of-no-interest to account for motion-related effects on blood-oxygen-level-dependent (BOLD). In line with our study aims, we created individual contrast maps for unpredictable threat (U-threat) > No-threat and unpredictable reward (U-reward) > No-reward for each person during first-level analysis.

These contrast maps were then first entered into a second-level one-way repeated measure analysis of variance (ANOVA) conducted using flexible factorial design in SPM, in order to examine the main effects of U-threat and U-reward across all participants (i.e. both samples). Next, to identify areas where significant activity was elicited by both U-threat and U-reward stimuli, a conjunction analysis (Nichols *et al.*, 2005) was performed within the framework of SPM using the two family-wise error (FWE) thresholded statistical maps identified in the above analysis that showed significant main effects of U-threat and U-reward (i.e. [U-threat > No-threat] AND [U-reward > No-reward]) in order to create an intersection map that revealed voxels with significant common activation. Finally, to identify areas where activity in the two tasks differed significantly, we performed a paired t-test comparing the main effects of U-threat and U-reward for each participant (i.e. [U-threat > U-reward], [U-reward > U-threat]). Regions were identified using built-in Talairach Atlas Labels in xjView (v.9.7; Human Neuroimaging Laboratory; Houston, TX) in conjunction with the Allen Brain Atlas (Allen Institute for Brain Science, Seattle, WA). Given the characteristics of our study sample, age (mean centered), gender, current or lifetime AUD and major depressive disorder (MDD) diagnoses were entered as covariates of no interest in all analyses to account for potentially confounding effects. In all second-level analyses, we considered activations that survived FWE whole-brain cluster extent correction at *P *< 0.05, with a cluster size greater than 20 contiguous voxels (volume > 160 mm^3^; Lieberman and Cunningham, 2009; Han and Glenn, 2018), as significant. These results were subsequently verified with permutations tests. Based on simulations (10 000 iterations) performed using 3dFWHMx and 3dClustSim with the autocorrelation function, correction at α < 0.05 is achieved with a voxel threshold of *P < *0.001 and cluster size of at least 106 contiguous voxels for U-threat (volume > 848 mm^3^) and 121 contiguous voxels for U-reward (volume > 968 mm^3^).

## Results

### Main effects of U-threat and U-reward

**Fig. 2.**
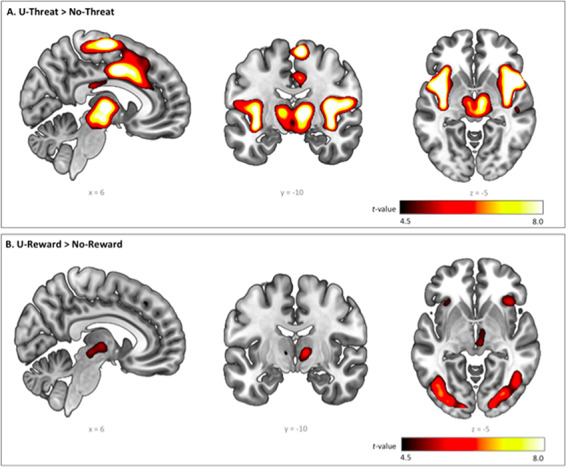
Whole-brain voxel-wise statistical *t* maps overlaid on a canonical brain, displaying significant activations at *P *< 0.05, family-wise error corrected (FWE), with a cluster size of 20 or more contiguous voxels, during (A) unpredictable threat > no threat and (B) unpredictable reward > no reward, across all participants. Color bars represent statistical *t*-scores.

Detailed neural activation elicited by U-threat and U-reward is presented in Figure 2 and Table 2. U-threat significantly activated the right insula (anterior and posterior), right supplementary motor area, left precuneus, left cerebellum, left dorsal anterior cingulate cortex (dACC) and left precentral gyrus. For U-reward, the whole-brain results yielded significant activations in regions previously associated with reward processing, including the right AIC, right ventral anterior nucleus of the thalamus (VA) and bilateral inferior frontal gyrus (IFG). Additional activations were found in bilateral fusiform gyrus.

**Table 2.** Main effects of the U-threat and U-reward task conditions

Region	MNI coordinates			Cluster (voxels)	Volume (mm^3^)	Z score
	*x*	*y*	*z*			
U-threat ≥ No-threat						
R insula (anterior and posterior)	36	−16	12	20 726	165 808	>8
R supplementary motor area	8	−10	66	6068	48 544	>8
L precuneus	−16	−46	66	81	648	6.16
L cerebellum	−34	−56	−34	228	1824	6.10
L anterior cingulate cortex (dorsal)	−14	−22	36	57	456	5.62
L precentral gyrus	−36	0	46	34	272	4.80
U-reward ≥ No-reward						
L fusiform gyrus	−32	−78	−16	2264	18 112	>8
R fusiform gyrus	34	−64	−14	1907	15 256	7.72
R inferior frontal gyrus	46	8	26	491	3928	6.04
R thalamus (ventral anterior nucleus)	10	−10	2	29	232	4.68
L inferior frontal gyrus	−38	8	28	36	288	4.56
R insula (anterior)	36	24	−2	20	160	4.45

*Note*: Reporting of all significant peak voxels at *P* < 0.05, family-wise error corrected (FWE), with a cluster size of >20 contiguous voxels.

L, left; MNI, Montreal Neurologic Institute; R, right; U, unpredictable.

### Common and differing activation for U-threat and U-reward

**Fig. 3.**
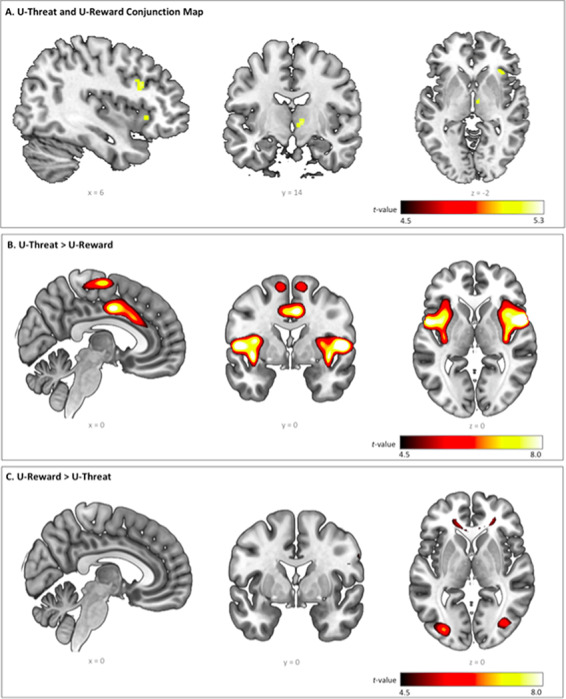
Whole-brain voxel-wise statistical *t* maps overlaid on a canonical brain, displaying significant activations at *P* < 0.05, family-wise error corrected (FWE), with a cluster size of 20 or more contiguous voxels, during (A) conjunction of unpredictable threat and unpredictable reward, (B) unpredictable threat > unpredictable reward and (C) unpredictable reward > unpredictable threat, across all participants. Color bars represent statistical *t*-scores.

The conjunction analysis revealed clusters of common activation in several brain regions, including the right AIC, right VA and right IFG. Significant differences between the two tasks were also identified; notably, activity in the right insula (anterior and posterior) and dACC was higher during the threat task (U-threat > No-threat) relative to the reward task (U-reward > No-reward). The opposite contrast revealed significantly higher activity primarily in the right fusiform and left middle occipital gyrus for reward relative to threat task. Figure 3 illustrates significant findings (see also Table 3). Of note, adjusting our analyses for covariates of no interest (i.e. age, gender, AUD and MDD diagnoses) did not change the results.

**Table 3.** Common (conjunction) and differing activation between U-threat and U-reward task conditions

Region	MNI coordinates			Cluster (voxels)	Volume (mm^3^)	Z score
	*x*	*y*	*z*			
Conjunction analysis						
R inferior frontal gyrus	46	16	22	240	1920	5.26
R thalamus (ventral anterior nucleus)	10	−10	2	29	232	4.68
R insula (anterior)	36	24	−2	20	160	4.45
Paired t-test (U-threat ≥ U-reward)						
R insula (anterior and posterior)	50	−26	22	5362	42 896	>8
L supramarginal gyrus	−56	−28	20	4578	36 624	>8
R postcentral gyrus	18	−42	64	1116	8928	>8
R anterior cingulate cortex (dorsal)	6	−2	40	1648	13 184	>8
L supplementary motor area	−10	−2	66	185	1480	5.93
L precuneus	−16	−46	66	39	312	5.18
R thalamus	16	−12	10	110	880	4.77
Paired t-test (U-reward ≥ U-threat)						
R fusiform gyrus	30	−60	−12	3658	29 264	7.46
L middle occipital gyrus	−32	−84	6	2930	23 440	7.38
R precentral gyrus	42	−14	62	49	392	5.23
R postcentral gyrus	60	−4	32	119	952	5.04
L postcentral gyrus	−60	−6	30	76	608	4.69

*Note*: Reporting of all significant peak voxels at *P* < 0.05, family-wise error corrected (FWE), with a cluster size of >20 contiguous voxels.

L, left; MNI, Montreal Neurologic Institute; R, right; U, unpredictable.

## Discussion

Temporally unpredictable stimuli influence behavior across species, as previously demonstrated for sequences of simple threats and rewards with fixed or variable onset (e.g. Bermudez and Schultz, 2014; Nelson *et al.*, 2015). Here, we showed that both temporally unpredictable threat and reward elicited activation in several common brain regions. Specifically, within the frontolimbic circuit, both processes engaged the right AIC. This finding is consistent with previous neuroimaging studies, which showed that the AIC is involved in processing of unpredictable threats (e.g. Shankman *et al.*, 2014), and more recently, unpredictable rewards as well (e.g. Gorka *et al.*, 2016). Thus, the AIC may be an important neural substrate involved in processing physiological and subjective responses to uncertainty (as a broadly defined construct).

Evidence indicates that the AIC integrates information about internal bodily states and salient environmental stimuli to produce interoceptive awareness and facilitate the generation of anticipatory emotional responses to positive or negative future outcomes (Craig, 2009). Fundamentally, during times of uncertainty, the AIC creates a subjective response to the question, ‘How is this going to feel?’. Related to this, the AIC uses information about interoceptive states to also perceive the passage of time, which is important given that we specifically manipulated the temporal predictability of uncertain stimuli (i.e. not knowing when the stimulus would occur). In the anticipation of uncertain stimuli, the AIC engages adaptive preparatory cognitive and behavioral resources that help an individual, avoid, minimize and cope with possible negative consequences. Dysfunction of the AIC may therefore lead to both (i) negatively biased perception of unpredictable threat, regardless of its true potential to confer harm (Paulus and Stein, 2006), and (ii) faulty appraisal of unpredictable reward (i.e. winning money) as distressing or over-arousing, which may diminish the hedonic and approach-eliciting aspects of reward (Nelson *et al.*, 2014). Therefore, chronic abnormal AIC activation may repeatedly impair appraisal of appetitive and aversive stimuli under uncertain conditions.

In addition to the AIC, both unpredictable threat and reward elicited activation in the right VA and right IFG, the two important auxiliary brain regions to the AIC that may play a role in responding to uncertainty. Studies have indicated that the VA has anatomical and functional connections with the rest of the thalamic nuclei and regions within the frontolimbic circuit, namely the basal ganglia and prefrontal cortices (McFarland and Haber, 2002; Grodd *et al.*, 2020), and is thus thought to be an important center for executive and motor functioning as well as reward and emotion processing (Xiao and Barbas, 2002, 2004; Child and Benarroch, 2013; Asami *et al.*, 2018; Wolff and Vann, 2019). Furthermore, in conjunction with other thalamic nuclei, the VA plays a role in both downstream and upstream pathways that carry viscerosensory information to be conveyed to the insula, cingulate cortex and prefrontal cortices. Increased VA activation during the anticipation of unpredictable threat and reward may therefore be related to its proposed role in processing of salient information to redirect attention and behavior (Cho *et al.*, 2013).

Research has also shown that IFG, a subregion of the lateral prefrontal cortex, may be another important node within the frontolimbic circuitry that may be implicated in emotion regulation (Cha *et al.*, 2016), as this region shows abnormal function in disorders with hyperarousal (anxiety) or hypoarousal (depression). More specifically, the IFG is important for the maintenance of the biological homeostasis, and its role is to effectively respond to salient emotional stimuli (appetitive or aversive) and efficiently return the neural system to baseline, and thus protect it from harm. Given the roles of the VA and IFG in maintenance of bodily homeostasis during the processing of uncertain stimuli, their dysfunction may lead to exacerbated aversive responding to uncertainty. However, this is speculative and remains to be further tested.

Although there are similarities in the neural circuitries underlying unpredictable threat and reward processing, there are also some notable differences. Relative to unpredictable reward, unpredictable threat recruited both the anterior and posterior clusters of the insular cortex, whereas unpredictable reward elicited activation only within the AIC cluster. Unpredictable threat also elicited greater activation (both in cluster size and in signal intensity) in the AIC compared with unpredictable reward (although see the ‘Limitations’ section below). The AIC is typically considered a key node involved in interoceptive awareness. However, posterior insula may also act as an integrative hub for information on subjective evaluation of internal and external states (Stephani *et al.*, 2011). Based on the current findings, however, posterior insula activation may be specific to the processing of unpredictable threat.

In addition to anterior and posterior insula, unpredictable threat, but not unpredictable reward, activated the dACC, which is also thought to contribute to the appraisal and expression of negative emotion and has a regulatory role with respect to limbic regions involved in generating emotional responses (Khalsa *et al.*, 2009; Etkin *et al.*, 2011). The connections between the dACC and the insula invite the hypothesis that the dACC plays a complementary role in generating a warning signal toward upcoming threat in order to encourage avoidance behavior (Klumpp *et al.*, 2012). The dACC is also a primary target of the mesocortical dopamine neurons (Paus, 2001), and therefore one might expect increased dACC response to rewarding or appetitive stimuli. However, we did not observe dACC activation during unpredictable reward. This may be due, in part, to the design of the NPU reward task. Most notably, unlike the MID task, this slot-machine task did not include a punishment condition in which participants lose money. This design distinction is important considering prior reports that showed greater dACC activation following trials that resulted in a loss relative to those that resulted in a gain (Gehring and Willoughby, 2002; Holroyd *et al.*, 2004).

Related to this, we also did not observe activation in the ventral striatum during the anticipation of unpredictable reward. This may be surprising given that previous studies often reported striatal activation during reward anticipation (Liu *et al.*, 2011). Nevertheless, prior work has also shown that anticipatory striatal activation may be contingent on an instrumental response (button press) and not just on imminent, potential reward delivery itself (Tricomi *et al.*, 2004; Bjork and Hommer, 2007). Thus, the lack of striatal response in the present study may be in part due to the task design being entirely passive (i.e. there were no behavioral performance component and no decision-making aspect).

On the other hand, unpredictable reward relative to unpredictable threat elicited more activation particularly in the visual cortex (i.e. right fusiform and left middle occipital gyrus), which could mean that unpredictable reward was perhaps more engaging.

The present study had several strengths including a relatively large sample size and the use of fMRI variants of the well-established NPU paradigm in order to independently examine the shared and unique neural correlates of temporally unpredictable threat and reward for the first time within the same sample of young adults. However, the present findings should be interpreted in light of several limitations. First, it is difficult to subjectively match the two reinforcers (i.e. mild electric shock and monetary reward) on emotional engagement. For each person, it is therefore possible that the observed differences in the neural response to unpredictable threat and reward may have been due to the difference in emotional intensity of the aversive and appetitive stimuli. The range and mean of the ITI (and the jitter) across the tasks were also different. One way to circumvent these problems in the future, and better equate the two tasks, may be to use reinforcers that are similar in nature (e.g. pairing primary reward [food, liquid] with primary threat [shock, aversive tone] or secondary reward [pleasant images, monetary gains] with secondary threat [negative images, monetary losses]) and tightly control the timing across paradigms. Related to this, future studies may also consider measuring additional indices of motivational engagement during the anticipatory periods in both shock and reward contexts (i.e. stimuli ratings, skin conductance) to control for in subsequent fMRI analyses and to ensure that the tasks worked as designed. Third, the present study examined neural response to temporally unpredictable threat and reward, but there are many ways one can manipulate uncertainty. Future studies should consider tasks that allow for a comparison of neural reactivity during the anticipation of unpredictable relative to predictable threats and rewards (such as NPU), which could involve manipulations of probability and/or magnitude. Doing so would ultimately allow for a better assessment of the neural circuitry underlying aversion/preference for uncertainty and its impact on behavior. Finally, adjusting our analyses for potential confounds (i.e. age, gender and AUD and MDD diagnoses) did not change the results. However, given our sample selection and characteristics, there still may be some other unmeasured confounding variable(s) that could not be accounted for in our analyses. Future studies are therefore needed to replicate and expand the present findings.

In conclusion, this study compared the neural response to temporally unpredictable threat and reward in a sample of young adults and found overlapping activation in the right AIC, right VA and right IFG. We also found preliminary evidence to suggest that some regions may be threat and reward specific. For instance, unpredictable threat may also recruit the posterior insula and dACC, while unpredictable reward may elicit increased activation in the visual cortex (i.e. fusiform and occipital gyrus). Taken together, the present findings suggest that although there is overlap in the neural circuitry underlying anticipation of temporally unpredictable threat and reward, these processes appear to be largely mediated by distinct circuits. However, more research is needed to corroborate these results using tasks that are better matched by design. Finally, future studies should also examine the generalizability of these findings to clinical populations and investigate how disruption of the neural activity within the aforementioned brain regions may contribute to psychopathology.

## Data Availability

The data that support the findings of this study are available from the corresponding author upon reasonable request.
